# Blood flow perfusion in visual pathway detected by arterial spin labeling magnetic resonance imaging for differential diagnosis of ocular ischemic syndrome

**DOI:** 10.3389/fnins.2023.1121490

**Published:** 2023-02-13

**Authors:** Yanan Chen, Xue Feng, Yingxiang Huang, Lu Zhao, Xi Chen, Shuqi Qin, Jiao Sun, Jing Jing, Xiaolei Zhang, Yanling Wang

**Affiliations:** ^1^Department of Ophthalmology, Beijing Friendship Hospital, Capital Medical University, Beijing, China; ^2^Department of Ophthalmology, Beijing Jishuitan Hospital, The Fourth Clinical Medical College of Peking University, Beijing, China; ^3^Department of Neurology, Beijing Tiantan Hospital, Capital Medical University, Beijing, China

**Keywords:** blood flow perfusion, visual pathway, arterial spin labeling, ocular ischemic syndrome, carotid stenosis, ocular neurodegeneration, optic atrophy

## Abstract

**Background:**

Ocular ischemic syndrome (OIS), attributable to chronic hypoperfusion caused by marked carotid stenosis, is one of the important factors that cause ocular neurodegenerative diseases such as optic atrophy. The current study aimed to detect blood flow perfusion in a visual pathway by arterial spin labeling (ASL) and magnetic resonance imaging (MRI) for the differential diagnosis of OIS.

**Methods:**

This diagnostic, cross-sectional study at a single institution was performed to detect blood flow perfusion in a visual pathway based on 3D pseudocontinuous ASL (3D-pCASL) using 3.0T MRI. A total of 91 participants (91 eyes) consisting of 30 eyes with OIS and 61 eyes with noncarotid artery stenosis-related retinal vascular diseases (39 eyes with diabetic retinopathy and 22 eyes with high myopic retinopathy) were consecutively included. Blood flow perfusion values in visual pathways derived from regions of interest in ASL images, including the retinal-choroidal complex, the intraorbital segments of the optic nerve, the tractus optics, and the visual center, were obtained and compared with arm-retinal circulation time and retinal circulation time derived from fundus fluorescein angiography (FFA). Receiver operating characteristic (ROC) curve analyses and the intraclass correlation coefficient (ICC) were performed to evaluate the accuracy and consistency.

**Results:**

Patients with OIS had the lowest blood flow perfusion values in the visual pathway (all *p* < 0.05). The relative intraorbital segments of optic nerve blood flow values at post-labeling delays (PLDs) of 1.5 s (area under the curve, AUC = 0.832) and the relative retinal–choroidal complex blood flow values at PLDs of 2.5 s (AUC = 0.805) were effective for the differential diagnosis of OIS. The ICC of the blood flow values derived from the retinal–choroidal complex and the intraorbital segments of the optic nerve between the two observers showed satisfactory concordance (all ICC > 0.932, *p* < 0.001). The adverse reaction rates of ASL and FFA were 2.20 and 3.30%, respectively.

**Conclusion:**

3D-pCASL showed that the participants with OIS had lower blood flow perfusion values in the visual pathway, which presented satisfactory accuracy, reproducibility, and safety. It is a noninvasive and comprehensive differential diagnostic tool to assess blood flow perfusion in a visual pathway for the differential diagnosis of OIS.

## Introduction

Marked stenosis or occlusion of the common or internal carotid arteries may cause ocular hypoperfusion (Lee et al., 2022) and/or cerebral hypoperfusion (Lineback et al., 2022). Ocular ischemic syndrome (OIS), attributable to chronic ocular hypoperfusion, is one of the important factors that cause ocular neurodegenerative diseases (Mester et al., 2009), such as optic atrophy (Battista et al., 2022). Ocular ischemic syndrome (OIS) describes ocular symptoms and signs attributable to ocular hypoperfusion caused by marked stenosis or occlusion of the common or internal carotid arteries (Terelak-Borys et al., 2012). It was first described by Hedges (1962), with their findings such as peripheral dot and blot hemorrhages and dilated retinal veins attributed to retinal hypoxia induced by carotid artery insufficiency (Casalino et al., 2017). It is a blinding and disabling disease (Hung and Chang, 2017) and has diverse clinical manifestations accompanied by asymptomatic injury (Mendrinos et al., 2010). It is usually asymptomatic but has potentially blinding abilities (Hung and Chang, 2017). The diagnosis of OIS can portend life-threatening cerebrovascular and cardiovascular complications (Mendrinos et al., 2010). The mortality rate of patients with OIS is 40% within 5 years from onset (Mills, 1989), and the most common causes of death are cardiac disease and stroke (Avery et al., 2019). The diagnosis of OIS is critical for saving visual function and improving the chances of survival.

The identification of the OIS and its various clinical manifestations presents an interdisciplinary challenge. In addition to OIS, there are also ischemic mechanisms present in retinal vascular diseases related to noncarotid artery stenosis, such as diabetic retinopathy (DR) and high myopia (HM) retinopathy (Steigerwalt et al., 2009). It was reported that the thinning of the choroid contributes more to the measured decreased chorioretinal perfusion than slowed arterial filling time (Vaghefi et al., 2017). Previous studies confirmed the ischemic mechanisms in DR and HM retinopathy. DR is a well-recognized ocular ischemic disease which is a microvascular complication of diabetes (Stolte and Fang, 2020). Mudaliar et al. reported that hyperglycemia causes retinal damage through complex metabolic pathways, leading to vascular damage, oxidative stress, capillary ischemia, and retinal tissue hypoxia. A growing body of evidence (Steigerwalt et al., 2009) suggests that HM is associated with decreased ocular blood flow (BF), the complications of which may contribute to severe visual loss. A recent study has shown that the aberrant blood perfusion of the cerebellum detected by ASL in patients with HM indicates a new understanding of brain abnormalities and brain plasticity (Wang et al., 2020).

Identifying a clinical distinction between OIS, which can potentially imply being affected by lethal disease and noncarotid artery stenosis-related retinal vascular disease, is essential and difficult. Therefore, reliable diagnostic biomarkers are needed. The traditional imaging modality for assessing ocular blood perfusion is fundus fluorescein angiography (FFA) (Terelak-Borys et al., 2012). Its invasive examination process relies on sodium fluorescein, an orange water-soluble dye, which is not applicable to all patients. Arterial spin labeling (ASL) magnetic resonance imaging (MRI) has been widely used in cerebrovascular disease (Scelsi et al., 2018). ASL allows magnetically labeled water protons from arterial blood as an endogenous diffusible tracer that disperses from the vascular system into neighboring tissues (Kitajima and Uetani, 2023). Voxel blood flow was quantified in mL/100 mL/min (Valentin et al., 2022). Anatomy and functionality are all important factors affecting tissue perfusion (Vaghefi et al., 2017). Therefore, we set the DR group in terms of arterial filling time and the HM group in terms of tissue volume.

This diagnostic test study was designed to detect blood flow perfusion in a visual pathway by ASL-MRI and explore an accurate, reproducible, and safe diagnostic tool for the differential diagnosis of OIS.

## Materials and methods

### Study design and participants

In this cross-sectional study, 91 participants (91 eyes) with retinal vascular diseases were prospectively and consecutively enrolled from November 2018 to November 2021. Participants included 30 patients with carotid artery stenosis (30 eyes with OIS) and 61 controls with noncarotid artery stenosis-related retinal vascular diseases (39 eyes with DR and 22 eyes with high myopic retinopathy).

The diagnostic criteria of OIS (Luo et al., 2018) are as follows: (1) the stenosis of the ipsilateral (to the affected eye) internal carotid artery (ICA) was >50%; (2) abnormal ocular symptoms and/or signs which cannot be explained by other ocular diseases; (3) FFA with the following signs: arm-choroidal circulation time>15 s, arm-retinal circulation time (ARCT) >18 s, and retinal circulation time (RCT) >11 s. The subjects that satisfied the first criterion and any of the two criteria in (2) or (3) led to a diagnosis of OIS (Lauria et al., 2020). The diagnostic criteria for DR (Fransen et al., 2002) are based on the international clinical DR severity grading standard established by the American Academy of Ophthalmology in 2002 (Nawaz et al., 2019; Flaxel et al., 2020). The diagnostic criteria for high myopic retinopathy are based on three key factors: atrophy (A), traction (T), and neovascularization (N), which is named the ATN classification system (Ruiz-Medrano et al., 2019).

The inclusion criteria were defined as follows: (1) patients with OIS; (2) patients with DR with severity greater than or equal to mild non-proliferative diabetic retinopathy; (3) patients with high myopic retinopathy with severity graded as A0-A4/T0-T3/N0-N2s.

The exclusion criteria were defined as follows: a history of other ocular diseases: glaucoma, uveitis, ocular trauma, or intraocular surgery; other types of retinal vascular diseases: retinal artery occlusion, retinal vein occlusion, retinal macroaneurysms, hypertensive retinopathy; MRI ineligibility (de Keizer and te Strake, 1986): claustrophobia or the presence of a cardiac pacemaker, joint replacement, or other implanted metal devices; MR images with visible artifacts; FFA ineligibility (Awan and Yang, 2006): hypersensitivity to sodium fluorescein and liver and kidney dysfunction; ocular diseases that diminished the quality of fundus image: serious cataract and vitreous hemorrhage. We excluded those who were implanted with metal devices (*n* = 1), had hypersensitivity to sodium fluorescein (*n* = 1), and whose MR and FFA images were of poor quality (*n* = 2).

The study was approved by the Medical Research Ethics Committee of Beijing Friendship Hospital, Capital Medical University (NO.2018-P2-185-02). All participants provided informed consent according to the Declaration of Helsinki.

### Clinical ophthalmic examination

**Figure 1:**
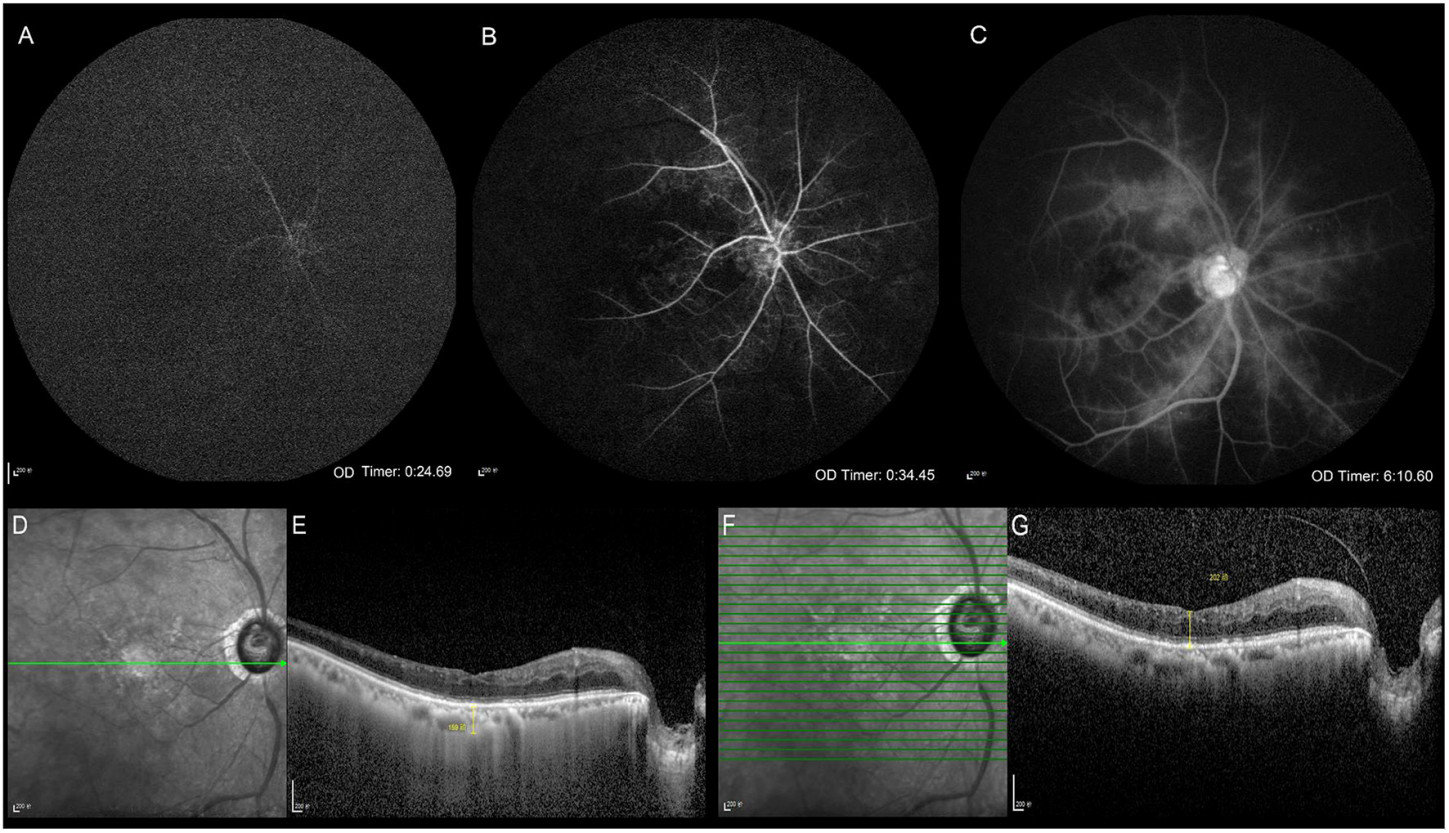
Clinical ophthalmic examinations. Fundus fluorescein angiography **(A–C)**. Optical coherence tomography **(D–G)**. The arm-retinal circulation time is 24.69 s showing the delayed retinal arierial filling **(A)**. The venous phase starts at 34.45 s, showing delayed retinal venous filling, which indicates that the retinal circulation time is 9.76 s **(B)**. Late retinal vascular staining **(C)**. The infrared image and the central macular choroidal thickness in enhanced depth imaging mode **(D, E)**. The infrared image and the central macular retinal thickness in conventional mode **(F, G)**.

All subjects underwent slit-lamp, optical coherence tomography (OCT, Heidelberg Spectralis), and FFA (Spectralis hra) examinations (Figure 1). OCT was used to measure the central macular retinal thickness in conventional mode. OCT measured the central macular choroidal thickness in the enhanced depth imaging (EDI) mode. FFA examinations were performed according to the requirements of the patient's condition. Allergy tests were carried out, and the subjects with negative results underwent a puncture of the median cubital vein and were injected with sodium fluorescein contrast medium. We collected the ARCT, RCT, capillary non-perfusion (NP) area, neovascularization (NV), retinal vascular staining, microaneurysms, and other fluorescein angiography signs. The same experienced technician completed each examination.

### ASL image acquisition

All subjects underwent a 3.0T MRI scan using a Philips Ingenia 3.0T scanner equipped with a 16-channel head coil. T1 and T2 weighted images, diffusion-weighted images, and 3D time-of-flight MR angiography images were obtained before the ASL sequence, and scanning time summed up to 20 min. Foam pads were placed at the sides of the subject's head to minimize head motion, and earplugs were used to reduce noise. During the MRI scan, subjects were instructed to close their eyes and stay relaxed to reduce eye movement.

The BF in the visual pathway was determined using the 3D pseudo-continuous ASL (3D-pCASL) technique, with the scan parameters as follows: gradient and spin echo sequence, post-labeling delay (PLD) = 1.5 s (repetition time [TR] = 3903 ms, echo time [TE] = 11 ms), PLD = 2.5 s (TR = 4903 ms, TE = 11 ms), bandwidth in echo-planar imaging = 2899.7 Hz, label distance = 90 mm, flip angle (FA) = 90°, slice thickness = 6 mm, number of slices = 20, slice gap = 0, slice orientation=transverse, field of view (FOV) = 240 × 240 mm, acquisition matrix = 64 × 64, number of excitations (NEX) = 3.

### ASL data quantification

**Figure 2:**
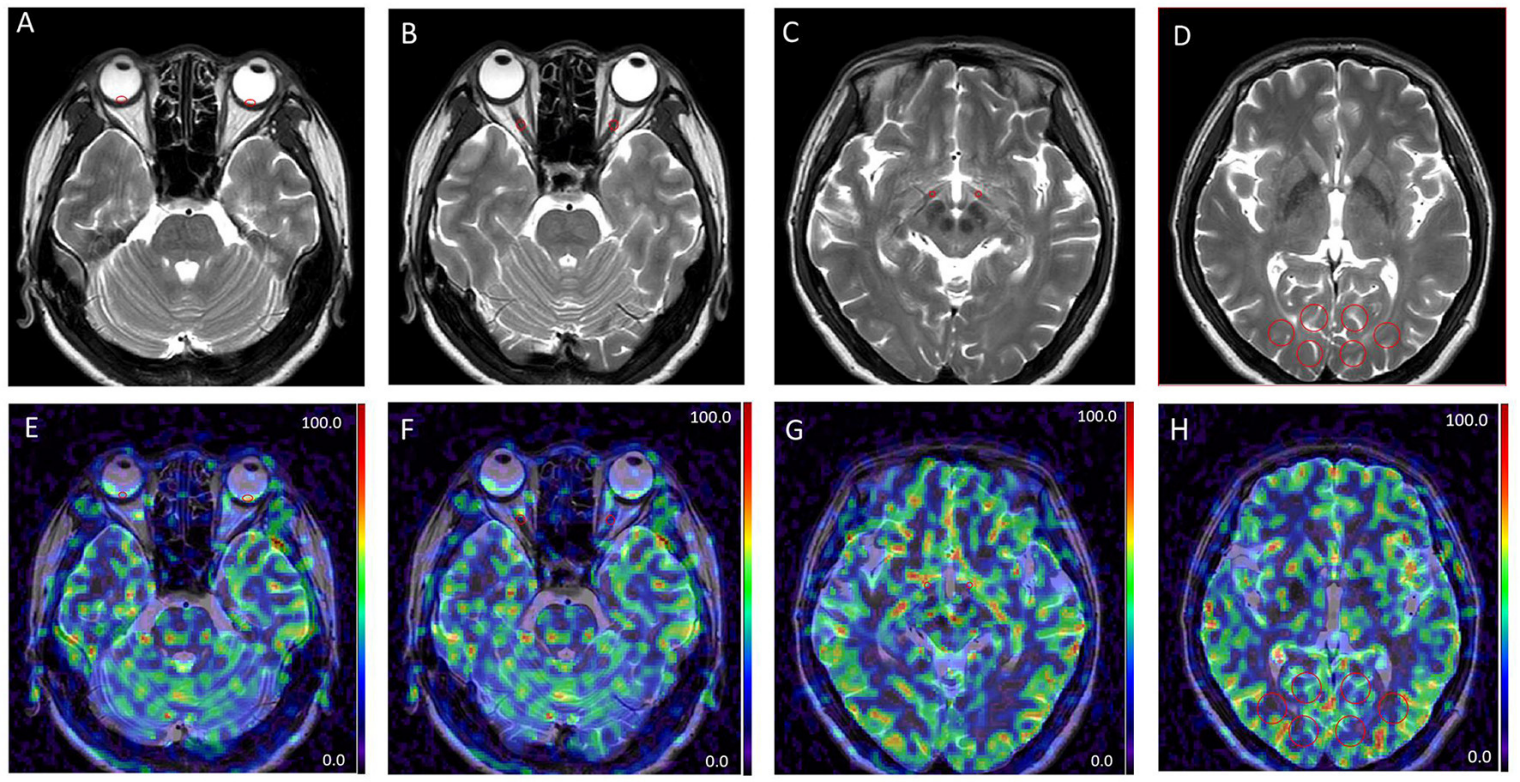
Examples of different patterns in MRI images. T2 weighted images **(A–D)**. Arterial spin labeling (ASL) images at post-labeling delay (PLD) of 1.5 s **(E–H)**. Regions of interest derived from the retinal-choroidal complex **(A, E)**; the intraorbital segments of the optic nerve **(B, F)**; the tractus opticus **(C, G)**; the visual center **(D, H)**; ROIs were all marked by red circles.

Blood perfusion maps were automatically obtained using the default process by the dedicated workstation (IntelliSpace Portal Release v.7.0.4.20175, Philips), and the data were derived from the blood perfusion maps. The regions of interest (ROIs) derived from the retinal-choroidal complex, the intraorbital segments of the optic nerve, the tractus opticus, and the visual center (Figure 2) were drawn by a neurologist (10 years of experience) and an ophthalmologist (10 years of experience), respectively, and clinical information was reviewed in a blinded fashion. The specific location of the retina-choroid complex, the orbital segment of the optic nerve, the optic tract, and the visual center were based on T1 and T2 weighted images. The unified criteria for drawing ROIs were as follows: The ROIs were all subrounded. The area of ROI of the retinal-choroidal complex, the intraorbital segments of the optic nerve, and the tractus opticus were 0.3 cm^2^; the area of ROI of the gyrus lingual, the cuneus, and the occipital lobe was 2 cm^2^, and the average BF value was taken as the BF of the visual center. The relative BF (rBF) value was defined as rBF = affected BF/healthy BF (Muir and Duong, 2011). The results of the measurements were retrieved from the two observers and calculated as the average value.

### Statistical analysis

Sample size considerations included the rarity of the OIS. This study hypothesizes that the area under the curve (AUC) of the BF perfusion values in a visual pathway is >0.5. Our pre-test showed that the AUC was >0.8. According to the following parameters, α= 0.05, β = 0.1, the power was calculated using PASS11.0 software, which was >90%, proving that the sample size was adequate.

Statistical analyses were performed using SPSS statistical software (version 26.0, SPSS) and GraphPad Prism software (version 6.0c, GraphPad Inc). Continuous variables were presented as mean ± standard deviation. A one-way ANOVA was used to analyze the differences among groups. Categorical variables were analyzed using Chi-square tests. Receiver operating characteristic (ROC) curve analyses were performed, and the AUC was applied to evaluate accuracy. A intraclass correlation coefficient (ICC) was performed to evaluate the consistency of BF values reported by the two observers; an ICC of >0.75 indicated satisfactory concordance. Statistical significance was accepted as a two-sided test with an alpha level of 0.05. A *P*-value of < 0.05 was considered statistically significant.

## Results

### Demographics and ocular characteristics

A total of 91 participants (mean [SD] age, 61.0 [10.0] years; 37 [40.7%] women) had 91 eyes with retinal vascular diseases, including 30 patients (30 eyes) with OIS after carotid artery stenosis and 61 controls with noncarotid artery stenosis-related retinal vascular diseases, which included 39 patients (39 eyes) with DR and 22 patients (22 eyes) with high myopic retinopathy. There were differences in age (*F* = 8.97, *p* < 0.001), with the predominant gender being male (χ2 = 16.54, *p* < 0.001) among the three groups. Subjects with OIS and high myopic retinopathy showed thinner central macular retinal thickness (*F* = 4.98, *p* = 0.009); subjects with high myopic retinopathy showed the thinnest central macular choroidal thickness (*F* = 42.65, *p* < 0.001). There were no significant differences in ARCT among the three groups (*F* = 1.40, *p* = 0.253). The differences among the three groups in the RCT were significant. The subjects with OIS showed the highest RCT values (*F* = 3.75, *p* = 0.027). The differences in the rates of capillary non-perfusion and neovascularization among the three groups were significant. The subjects with DR showed the highest rates of capillary non-perfusion (χ^2^ = 27.66, *p* < 0.001) and neovascularization (χ^2^ = 22.00, *p* < 0.001). The demographics and clinical characteristics of each group are represented in Table 1.

**Table 1:** Demographics and ocular characteristics.

**Variable**	**Total (*n =* 91)**	**OIS (*n =* 30)**	**DR (*n =* 39)**	**HM (*n =* 22)**	***p-*value**
Gender, female/male, n (%)	37(40.7)/54(59.3)	5(16.7)/25(83.3)	16(41.0)/23(59.0)	16(72.7)/6(27.3)	< 0.001
Age, years, mean (SD)	61.0 (10.0)	66.6 (8.3)	59.3 (7.8)	56.3 (12.3)	< 0.001
**OCT**					
Central macular retinal thickness, μm, mean (SD)	265.90 (122.81)	223.47 (30.12)	309.33 (142.75)	242.50(145.31)	0.009
Central macular choroidal thickness, μm, mean (SD)	211.84 (93.55)	243.41 (61.80)	252.61 (73.53)	94.48 (60.43)	< 0.001
**FFA**					
ARCT, seconds, mean (SD)	18.00 (5.51)	19.30 (6.60)	17.61 (4.68)	16.87 (5.12)	0.253
RCT, seconds, mean (SD)	3.87 (4.37)	5.60 (7.18)	3.06 (1.19)	2.89 (0.89)	0.027
Capillary non-perfusion, n (%)	22 (24.2)	2 (6.7)	20 (51.3)	0 (0)	< 0.001
Neovascularization, n (%)	24 (26.4)	3(10)	20(51.3)	1(4.5)	< 0.001

OIS, ocular ischemic syndrome; DR, diabetic retinopathy; HM, high myopia; OCT, optical coherence tomography; FFA, fundus fluorescein angiography; ARCT, arm-retinal circulation time; RCT, retinal circulation time; SD, standard deviation.

p-values for comparisons among OIS, DR, and HM.

p < 0.05 was considered statistically significant.

### ASL characteristics based on ROI analysis

**Figure 3:**
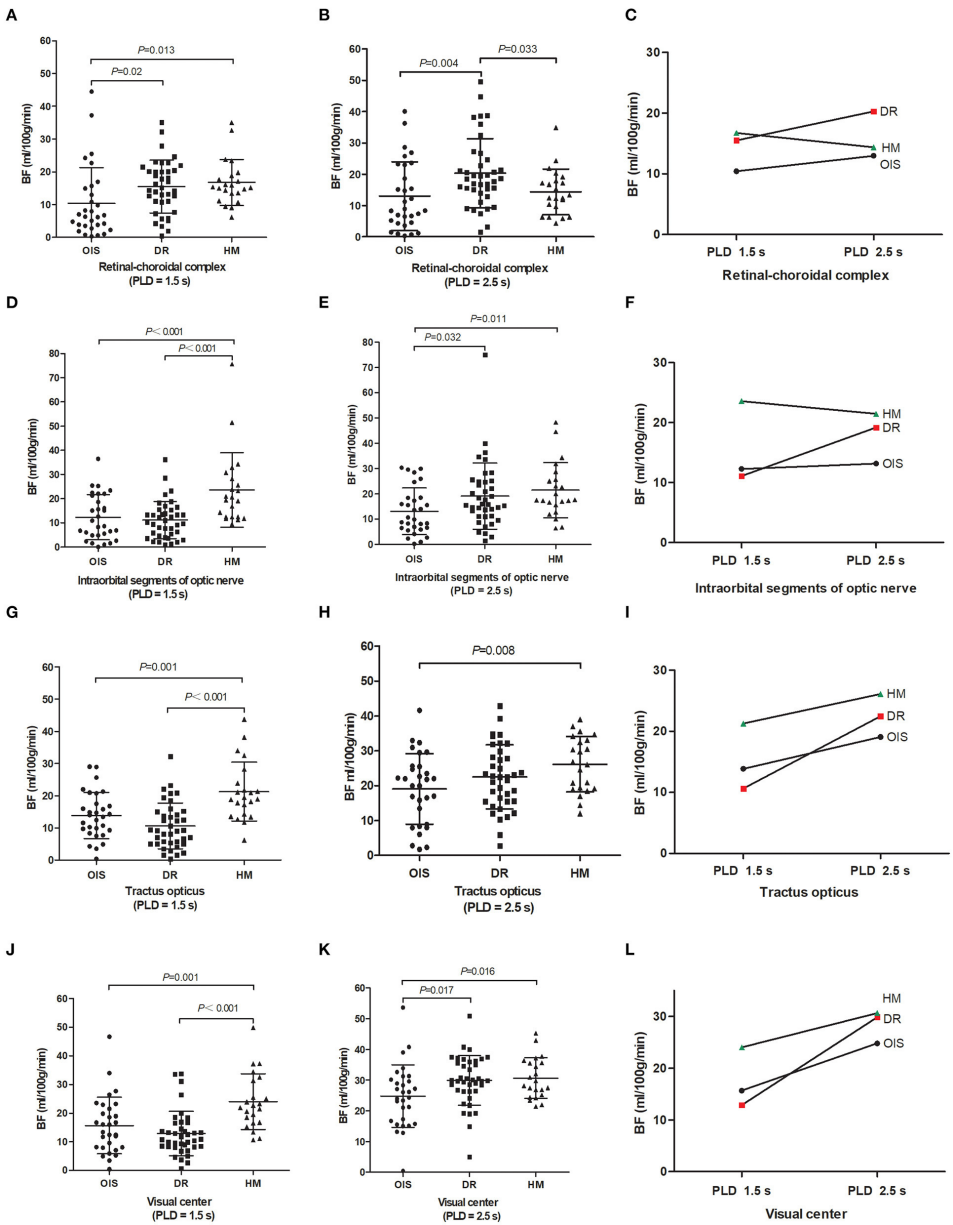
Between-group comparison. Magnetic resonance imaging metrics for each subject are shown as raw data with lines for mean and standard deviation **(A, B, D, E, G, H, J, K)**. Images showing blood flow perfusion values changed from PLD 1.5–2.5 s **(C, F, I, L)**. BF, blood flow; PLD, post-labeling delay; OIS, ocular ischemic syndrome; DR, diabetic retinopathy; HM, high myopia.

There were significant differences among the three groups in detectable BF values of the visual pathway at PLDs of 1.5 and 2.5 s, including the BF values of the retinal–choroidal complex (*F* = 4.065*, p* = 0.020*; F* = 4.923*, p* = 0.009), the intraorbital segments of the optic nerve (*F* = 10.873, *p* < 0.001; *F* = 3.907, *p* = 0.024), the tractus opticus (*F* = 13.617, *p* < 0.001; *F* = 3.738, *p* = 0.028), and the visual center (*F* = 11.057, *p* < 0.001; *F* = 4.012, *p* = 0.022) (Table 2). Subjects with OIS had the lowest BF perfusion values in the visual pathway at PLD of 1.5 and 2.5 s among the three groups (all *p* < 0.05). Subjects with DR were presented with lower BF perfusion values in the intraorbital segments of the optic nerve, the tractus opticus, and the visual center at a PLD of 1.5 s (all *p* < 0.05). Subjects with high myopic retinopathy were presented with lower BF perfusion values in the retinal–choroidal complex at a PLD of 2.5 s (all *p* < 0.05). Most of the perfusion values in the visual pathway increased from PLD 1.5 s to PLD 2.5 s (Figure 3).

**Table 2:** ASL characteristics based on ROI analysis.

**Variable**	**Total (n= 91)**	**OIS (*n =* 30)**	**DR (*n =* 39)**	**HM (*n =* 22)**	***p* value**
**ASL: BF (PLD** = **1.5 s)**					
Retinal–choroidal complex, ml/100 g/min, mean (SD)	14.15 (9.17)	10.43 (10.88)	15.52 (8.07)	16.77 (6.99)	0.020
Intraorbital segments of optic nerve, ml/100 g/min, mean (SD)	14.52 (11.62)	12.29 (9.33)	11.12 (7.73)	23.59 (15.36)	< 0.001
Tractus opticus, ml/100 g/min, mean (SD)	14.27 (8.69)	13.87 (7.20)	10.62 (7.13)	21.28 (9.14)	< 0.001
Visual center, ml/100 g/min, mean (SD)	16.50 (9.93)	15.68 (9.91)	12.87 (7.72)	24.03 (9.70)	< 0.001
**ASL: BF (PLD** = **2.5 s)**					
Retinal–choroidal complex, ml/100 g/min, mean (SD)	16.46 (10.66)	12.99 (10.95)	20.29 (11.03)	14.40 (7.22)	0.009
Intraorbital segments of optic nerve, ml/100 g/min, mean (SD)	17.74 (11.77)	13.15 (9.18)	19.18 (13.09)	21.46 (10.91)	0.024
Tractus opticus, ml/100 g/min, mean (SD)	22.23 (9.52)	19.05 (10.10)	22.48 (9.21)	26.12 (7.97)	0.028
Visual center, ml/100 g/min, mean (SD)	28.40 (8.81)	24.81 (10.15)	29.87 (8.09)	30.67 (6.65)	0.022

ASL, arterial spin labeling; ROI, region of interest; OIS, ocular ischemic syndrome; DR, diabetic retinopathy; HM, high myopia; BF, blood flow; PLD, postlabeling delay; SD, standard deviation.

*p*-values for comparisons among OIS, DR, and HM.

*p* < 0.05 was considered statistically significant.

### Accuracy of ASL in the differential diagnosis of OIS

**Figure 4:**
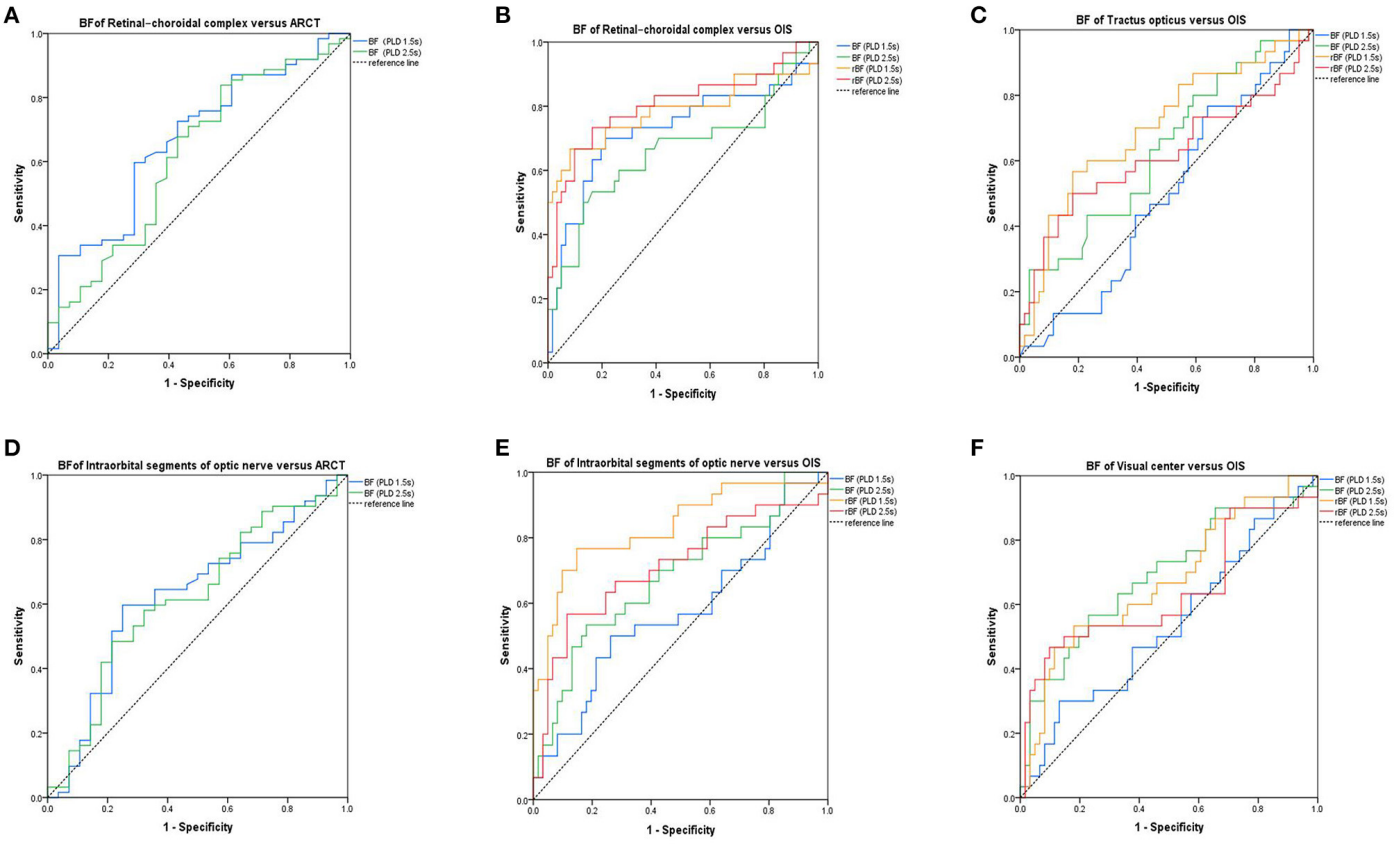
Receiver operating characteristic curves. The area under curve (AUC) showing the accuracy of the values of blood flow perfusion in the visual pathway, identified using arterial spin labeling (ASL) for diagnosis of the delayed retinal arterial filling **(A, D)**. The AUC shows the accuracy of the values of blood flow perfusion in the visual pathway, identified using ASL for diagnosis of OIS **(B, C, E, F)**. BF, blood flow; rBF, relative blood flow; ARCT, arm-retinal circulation time; OIS, ocular ischemic syndrome.

The accuracy of ASL in the diagnosis of OIS was evaluated using the ROC curve analysis (Figure 4). The BF values of the retinal–choroidal complex at a PLD of 1.5 s [AUC:0.669; 95% confidence interval (CI) 0.55–0.79; *p* = 0.01] were estimated by comparison with the ARCT of the gold standard FFA-based diagnosis of delayed retinal arterial filling. The relative intraorbital segments of optic nerve BF values at PLDs of 1.5 s (AUC:0.832; 95%CI 0.74–0.93; *p* < 0.001), with a cutoff point of 0.79 (sensitivity:76.7%; specificity:85.2%), and the relative retinal–choroidal complex BF values at PLDs of 2.5 s (AUC:0.805; 95%CI 0.70–0.92; *p* < 0.001), with a cutoff point of 0.78 (sensitivity:73.3%; specificity:83.6%), were effective predictors for the differential diagnosis of OIS.

### Concordance between observers in ASL

There was concordance between the two observers, with an ICC of 0.932 (95% CI 0.897–0.955, *p* < 0.001) at PLDs of 1.5 s and 0.974 (95%CI 0.956–0.984, *p* < 0.001) at PLDs of 2.5 s for the retinal–choroidal complex. The ICC of the BF values of the intraorbital segments of optic nerve BF between the two observers was 0.972 (95%CI 0.956–0.982, *p* < 0.001) at PLDs of 1.5 s and 0.984 (95%CI 0.974–0.990, *p* < 0.001) at PLDs of 2.5 s. The ICC of the BF values of the optic tract and the visual center at PLDs of 1.5 s and PLD of 2.5 s were all more than 0.984 (all *p* < 0.001).

### Safety of ASL and FFA

Of the 91 subjects, two patients felt uncomfortable due to the claustrophobic space of the MRI, and three patients developed a mild rash due to the sodium fluorescein contrast agent. The adverse reaction rates of ASL and FFA were 2.20 and 3.30%, respectively. There was no significant difference in the safety between ASL and FFA (*p* < 0.001), but ASL was noninvasive and, independent of contrast media, showed better convenience.

## Discussion

In our study, subjects with OIS tended to be older, with a male predominance, keeping with characteristics described in the literature (Xiang and Zou, 2020). The characteristics of the disease made complete matching impossible. Previous studies (Vaghefi et al., 2017) showed that, in addition to the rate of BF, the volume of the vascular tissue may be one of the important factors that will influence the perfusion of the eye in ASL. Central macular retinal and choroidal thickness measured by EDI-OCT can be the surrogate biomarker of the vascular tissue, which is known to decrease with increasing age (Ikuno et al., 2011). Our study showed that subjects with OIS and high myopic retinopathy showed thinner central macular retinal thickness compared to subjects with DR, which was consistent with the characteristics of the disease reported in the earlier literature (Brito et al., 2015). Another finding was that subjects with HM presented with the thinnest central macular choroidal thickness compared with the other two groups, which was in keeping with previous studies (Fang et al., 2019). The blood supply of the visual pathway is from the ophthalmic artery, the middle cerebral artery, and the posterior cerebral artery (Abhinav et al., 2020). A study (Dan et al., 2019) assessed resting cerebral blood flow changes in patients with retinitis pigmentosa using a pseudo-continuous ASL and found that altered cerebral BF may cause trans-synaptic retrograde degeneration of the visual pathway in patients with retinitis pigmentosa. We also found some interesting results: The subjects with DR were presented with lower BF perfusion values in the intraorbital segments of the optic nerve, the tractus opticus, and the visual center at PLDs of 1.5 s. A previous study (Wong et al., 2020) showed the association between DR and an increased risk of stroke, which indicated that the larger cerebrovascular implications are caused by the microvascular pathology inherent to DR. Therefore, we speculated that the BF perfusion of the visual pathway in patients with DR was affected by systemic diseases. The results of the present study also showed that the subjects with high myopic retinopathy presented with lower BF perfusion values in the retinal–choroidal complex at PLDs of 2.5 s, which further confirmed that the volume of the vascular tissue is another factor that will affect the perfusion of the posterior pole in ASL. ASL is used to evaluate the tissue perfusion rate. Tissue perfusion—the exchange of water and nutrients with tissues—occurs over the entire length of capillaries (Zhu et al., 2022). ASL basically “tracks” the water molecules in the blood from the arterial cavity to the tissue capillary bed and treats the water molecules as a freely diffusible tracer. ASL can easily occur through magnetization reversal or saturation of blood and water molecules in the blood supply artery along the *Z*-axis (Moran et al., 2022). After labeling, the time to wait for the blood to enter the tissue is called the PLD time or the reversal time of some specific ASL technology. Select the delay time so that the image can be obtained, ideally when the water molecules and tissues are magnetized and exchanged. Arterial blood labeling is realized through the combination of pulse and gradient to reverse the longitudinal magnetization of blood-water protons (Iutaka et al., 2023).

The accuracy of ASL perfusion evaluation is essential to diagnosing OIS. As the primary cause of OIS, the stenosis or occlusion of the common or internal carotid arteries is easy to ignore, and it is a necessary condition for diagnosing OIS (Mendrinos et al., 2010). The most specific (but not the most sensitive) fluorescein angiography sign of OIS is prolonged retinal filling time, known as ARCT, which is present in approximately 60% of patients with OIS (Terelak-Borys et al., 2012). The most sensitive (but not the most specific) fluorescein angiography sign of OIS is prolonged RCT, which is present in 95% of patients with OIS (Brown and Magargal, 1988). The BF values of the retinal–choroidal complex at PLDs of 1.5 s were estimated by comparison with the ARCT of the gold standard FFA-based diagnosis of delayed retinal arterial filling in our study. The result of an AUC of 0.669 was not satisfactory. However, the results of the rBF value were satisfactory. In previous studies of cerebral blood flow perfusion, the relative cerebral perfusion value (Iutaka et al., 2023) was more concerning than the absolute value (Salisbury et al., 2022). However, a delayed arterial filling time is not diagnostic for ocular ischemia (Hung and Chang, 2017). In Vaghefi's report (Vaghefi et al., 2017), they attempted to quantify the chorioretinal blood perfusion in patients with a clinical diagnosis of retinal ischemia using ASL. They speculated that ocular ischemia may be due to tissue volume and arterial flow, but only four participants without blood perfusion of the visual pathway were evaluated in their study.

The reproducibility of ASL perfusion evaluation is necessary for clinical application in diagnosing OIS. In our study, the ICC of the BF values derived from the retinal–choroidal complex and the intraorbital segments of the optic nerve between the two observers at PLDs of 1.5 and 2.5 s showed satisfactory concordance. A previous study (Khanal et al., 2019) demonstrated the high intraday and interday repeatability in the quantitative ASL-MRI measurements of retinal–choroidal complex blood perfusion. However, their study did not evaluate other blood perfusion values in the visual pathway. When we suspect that the patient has ocular hypoperfusion, we should combine ASL with FFA to make a comprehensive judgment. When ASL is applied in the eye, the blood perfusion in the posterior part of the eye will be measured, and the low perfusion of the visual pathway can be presented, which will help to understand the factors affecting the changes in the blood perfusion of the visual pathway and the changes in the blood perfusion of the eye caused by carotid artery stenosis.

The safety and convenience of the clinical application of ASL in the differential diagnosis of OIS may be attractive to ophthalmologists compared with traditional ophthalmic examinations. However, FFA is the gold standard for diagnosing retinal vascular diseases. The limitations of FFA in itself affect the clinical application. In our study, two patients felt uncomfortable due to the claustrophobic space of the MRI, and three patients developed a mild rash due to the sodium fluorescein contrast agent. Although there was no significant difference in the safety of ASL and FFA, ASL was noninvasive and showed more advantages, independent of contrast media.

## Conclusion

In conclusion, 3D-pCASL showed the participants with OIS had lower blood flow perfusion values in the visual pathway, which presented satisfactory accuracy, reproducibility, and safety. It is a noninvasive and comprehensive diagnostic tool to assess blood flow perfusion in a visual pathway for the differential diagnosis of OIS.

### Limitations

The limitations of this study are as follows. The spatial resolution of images is larger than the areas of intraorbital ROIs and the tractus opticus, which are determined by the size of the study organ. ASL of white matter, particularly small white matter tracts, has always been problematic, even when this study used the contralateral side as an internal reference. However, we attempted to include complete clinical data for analysis to explore OIS's noninvasive differential diagnosis strategy.

## Data availability statement

The original contributions presented in the study are included in the article/supplementary material, further inquiries can be directed to the corresponding authors.

## Ethics statement

The studies involving human participants were reviewed and approved by the Medical Research Ethics Committee of Beijing Friendship Hospital, Capital Medical University (NO.2018-P2-185-02). The patients/participants provided their written informed consent to participate in this study.

## Author contributions

XZ and YW supervised the present study. YC and XF performed the analysis and wrote the manuscript. YH, LZ, XC, SQ, and JS helped to collect the clinical data. JJ contributed to the image processing. All authors contributed to the article and approved the submitted version.
